# Cross-cultural adaptation of Family Satisfaction with Care in the Intensive Care Unit 24R - Brazilian version

**DOI:** 10.1590/0034-7167-2024-0273

**Published:** 2025-12-12

**Authors:** Josiele de Lima Neves, Eda Schwartz, Lílian Moura de Lima Spagnolo, Eraldo Schunk Silva, Andrieli Daiane Zdanski de Souza, Fernanda Lise

**Affiliations:** I Universidade Federal de Pelotas. Pelotas, Rio Grande do Sul, Brazil; II Hospital de Clínicas de Porto Alegre. Porto Alegre, Rio Grande do Sul, Brazil; III Fundação Universidade Rio Grande. Rio Grande, Rio Grande do Sul, Brazil; IV Universidade Estadual de Maringá. Maringá, Paraná, Brazil

**Keywords:** Family, Satisfaction, Intensive Care Units, Validation Study, Nursing., Familia, Satisfacción, Unidades de Cuidados Intensivos, Estudio de Validación, Enfermería.

## Abstract

**Objectives:**

to cross-culturally adapt and validate the content of Family Satisfaction with Care in the Intensive Care Unit 24R for use in Brazil.

**Methods:**

methodological research. Translation, translation consensus, equivalence assessment, back-translation, back-translation consensus, and pre-test were performed. Seven experts assessed translation equivalence and content validity. Applicability, item comprehension, and internal consistency were verified in a pre-test with 30 family members. Data were analyzed using the Content Validity Index, semantic assessment, correlation (r) between items, and Cronbach’s alpha.

**Results:**

linguistic adjustments were made to make the items understandable. The Content Validity Index was 0.86, Cronbach’s alpha was 0.93, and all items presented a correlation ≥0.30. Semantic assessment obtained more than 85% approval.

**Conclusions:**

the adapted questionnaire had its content validated, proving to be reliable for assessing psychometric properties. It showed potential to contribute to assessing family satisfaction in intensive care in Brazil, in order to identify aspects that need to be improved as well as those that should be maintained and strengthened.

## RESUMEN

**Objetivos:**

realizar la adaptación transcultural y validar el contenido del *Family Satisfaction with Care in the Intensive Care Unit* 24R para su uso en Brasil.

**Métodos:**

investigación metodológica. Se realizaron la traducción, el consenso de la traducción, la evaluación de equivalencias, la retrotraducción, el consenso de la retrotraducción y una prueba previa. Siete expertos evaluaron las equivalencias de la traducción y la validez de contenido. La aplicabilidad, la comprensión de los ítems y la consistencia interna se verificaron en una prueba previa con 30 familiares. Los datos se analizaron mediante el Índice de Validez de Contenido, la evaluación semántica, la correlación (r) entre ítems y el alfa de Cronbach.

**Resultados:**

se realizaron ajustes lingüísticos para facilitar la comprensión de los ítems. El Índice de Validez de Contenido fue de 0,86, el alfa de Cronbach de 0,93 y todos los ítems presentaron una correlación ≥0,30. La evaluación semántica obtuvo una aprobación superior al 85%.

**Conclusiones:**

el cuestionario adaptado fue validado en su contenido, demostrando ser fiable para evaluar las propiedades psicométricas. Demostró potencial para contribuir a la evaluación de la satisfacción familiar en cuidados intensivos en Brasil, con el fin de identificar aspectos que requieren mejora, así como aquellos que deben mantenerse y fortalecerse.

## INTRODUCTION

Admission of patients to the Intensive Care Unit (ICU) usually involves close family members. Moreover, the term “family members” includes people closely involved in the process of admission and stay of patients in the ICU, such as spouses, children or parents, but also friends and people close to patients^(1)^. To improve the quality of care provided to families, it is important to assess family members’ satisfaction with the care and support they received to identify areas that need improvement^(2)^.

In this context, to assess family satisfaction, it is necessary to use reliable, validated and widely used instruments that can contribute to healthcare management. In this regard, the Brazilian National Policy for Humanization of Healthcare and Management in the SUS, known as HumanizaSUS, contributes to improving care management, since, being inclusive and resolute, it offers innovations in management practices, strengthening the relationship between patient, team and family^(3)^.

Although the assessment of family satisfaction with care, environment, staff and decision-making is an incipient topic in the Brazilian reality, progress is evident in scientific production. In 2018, in Brazil, a reliable version in Brazilian Portuguese^(4)^ of the Canadian questionnaire Family Satisfaction with Care in the Intensive Care Unit (FS-ICU 24) was presented^(5)^. In the same year, the authors of the original instrument made available the revised version of FS-ICU 24R, in which modifications were made to some items and the letter “R” for revised was added after the acronym^(6)^.

In literature, it is identified that FS-ICU 24^(5)^ is recognized for supporting the team and family in decision-making and is widely used in international studies, having been adapted in countries such as Brazil^(4)^, Germany^(7)^, South Korea^(8)^, China^(9)^, Iran^(10)^, Norway^(11)^, Thailand^(12)^, and Turkey^(13)^. The need to validate the content and semantics of the new version of FS-ICU 24R was identified to make items understandable and appropriate, allowing the instrument to be safely validated in the Brazilian context and thus contribute to advances in care management, offering an updated, reliable and valid version to be tested with Brazilian families in ICUs.

## OBJECTIVES

To cross-culturally adapt and validate the content of FS-ICU 24 for use in Brazil.

## METHODS

### Ethical aspects

Before the research was conducted, authorization was granted, via email, by the author who holds the rights to the questionnaire. All procedures performed respected ethical principles, and the research was approved by the Research Ethics Committee.

### Study design

This is a methodological study of cross-cultural adaptation of the revised version^(6)^ of FS-ICU 24^(5)^ from English to Brazilian Portuguese, developed between February 2019 and September 2021. For its development, the checklist for qualitative studies was followed.

FS-ICU 24R consists of 24 items that allow the assessment of family satisfaction with care, the environment, the team and their participation in decision-making^(6)^. Among them, item 2 is subdivided into 2a, 2b and 2c, which operationally adds two items. In addition, the questionnaire includes the assessment of relatives of those who died, through items 25, 26 and 27. Therefore, 29 items will be operationally assessed. Originally, items are distributed into two domains: satisfaction with care; and family satisfaction with decision-making around care of critically ill patients^(6,14)^.

Response options for items 1 through 20 are presented on a five-point Likert scale ranging from 1 (very dissatisfied) to 5 (completely satisfied), plus the option not applicable (N/A). Between items 21 and 27, response alternatives are organized into sets of five ideas for the choice of the one that best expresses the opinion. For both, the higher the score obtained, the more favorable the family member’s satisfaction^(14)^.

The questionnaire reliability was verified by internal consistency, by applying Cronbach’s alpha coefficient, testing the change in its value according to the hypothetical removal of items, with verification of correlations between responses. Values between 0.7 and 0.9 were considered ideal, and in the analysis of the correlation of items, items with correlation above 0.3 were considered adequate^(15)^.

**Figure 1:**
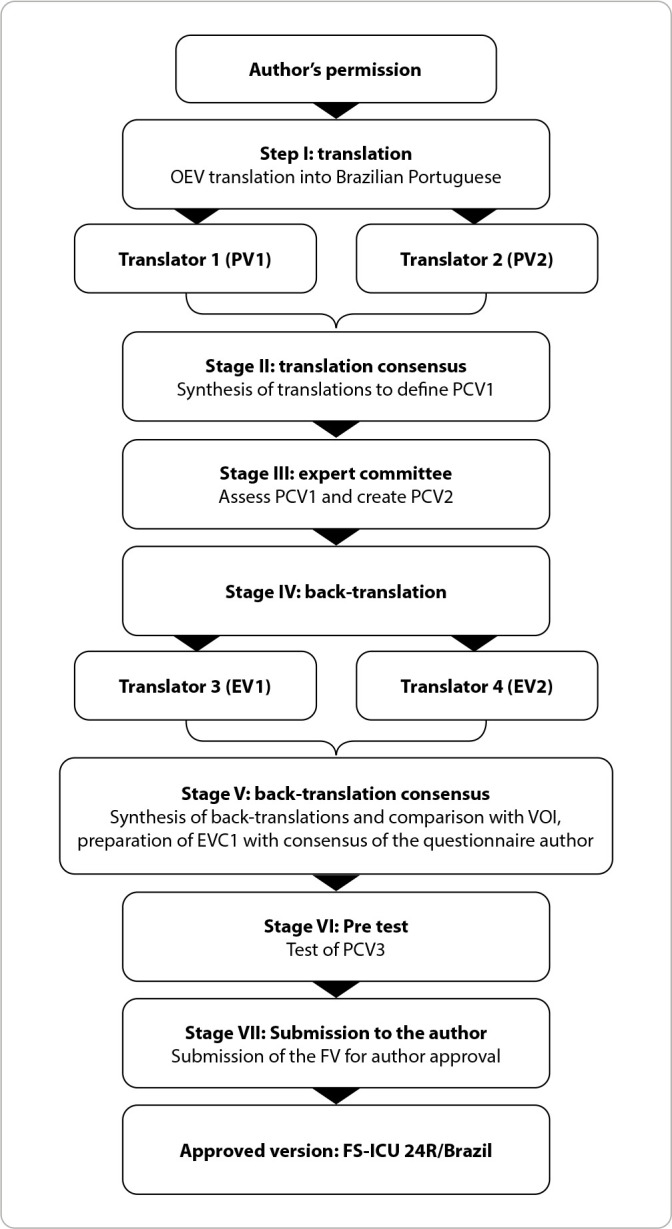
Flowchart of the methodological trajectory of cross-cultural adaptation of Family Satisfaction with Care in the Intensive Care Unit 24R for use in Brazil, Pelotas, Rio Grande do Sul, Brazil, 2022

The translation and adaptation process were based on Guidelines for the Process of Cross-Cultural Adaptation of Self Report Measures^(16)^ recommendations, with changes in the order of the stages^(17)^ and in line with Brazilian studies^(18,19)^. It comprised the following stages (Figure 1): I translation into Brazilian Portuguese; II Portuguese version consensus; III assessment by an expert committee; IV back-translation into the original language; V English version consensus and comparison with the original version; VI pre-test; and VII sending the original version to the author.

*OEV - original English version; PV1 - Portuguese version translator 1; PV2 - Portuguese version translator 2; PCV1 - Portuguese version consensus 1; PCV2 - Portuguese version consensus 2; EV1 - English version translator 1; EV2 - English version translator 2; EVC - English version consensus; PCV3 - Portuguese version consensus 3; FV - final version.*

### Field study

#### Stages 1 and 2 - Translations and consensus of Portuguese versions

Stage I, which corresponds to translation into Brazilian Portuguese, was carried out independently by two Brazilian translators (translator 1 and translator 2), who received an invitation letter requesting a translation that preserved semantic equivalence of original items. The inclusion criteria were being Brazilian, having a command of English and extensive knowledge of Canadian culture. Each translator produced a translation certificate and a report of Portuguese versions. After obtaining the two versions, Portuguese version 1 (PV1) and Portuguese version 2 (PV2), stage II was carried out, in which the two translators met in a meeting to discuss and summarize the translated versions, resulting in Portuguese consensus version 1 (PCV1).

#### Stage 3 - Analysis by an expert committee

In stage III, the analysis was carried out by a committee composed of seven experts. The members of this committee were invited based on some criteria, which all met: being a healthcare professional, experienced with the method of this research and/or with English and/or with intensive care. This stage was developed in two stages: 1) with seven experts, semantic, idiomatic, cultural and conceptual equivalences between the translated and synthesized version (PCV1) were verified and compared with the original version. Thus, the proportion or percentage of agreement among experts was verified through Content Validity Index (CVI). The adequacy of each item varied between -1 (not equivalent), 0 (unable to assess/do not know) and +1 (equivalent), according to a similar study^(20)^. In the second stage, to ensure consensus version comprehension and reliability, the items and domains with the lowest percentage of equivalences were simultaneously reassessed in a virtual meeting by a translator from the first stage, two nurses specialized in intensive care and with expertise in instrument adaptation and validity, and the researchers as mediators. After adjustments, the Portuguese consensus version 2 (PCV2) was submitted to back-translation.

#### Stage 4 - Back-translation into the original language

In stage IV, back-translation, two Canadian translators (translator 3 and translator 4), with extensive command of Brazilian Portuguese, knowledgeable about Brazilian culture and resident in Brazil, were invited to translate PCV2, generating two independent back-translations, called English versions 1 and 2 (EV1 and EV2).

#### Stage 5 - English version consensus and comparison with the original version

Stage V consists of ensuring that the translated version reflects the same content as the original version. A virtual meeting was organized to assess equivalences between the original questionnaire and back-translations, with the participation of one of the translators of back-translation, a nurse with extensive knowledge of English and methodological studies, mediated by the researchers. After the meeting, the English version consensus 1 (EVC1) was sent for review by the questionnaire author. After adjustments, the version was translated into Portuguese and named Portuguese consensus version 3 (PCV3).

#### Stage 6 - Pre-testing with the target population

In stage VI, pre-test was carried out, which consisted of applying PCV3 to the target population, which met the following criteria: being over 18 years old, being fluent in Brazilian Portuguese, having visited patients (hospitalized for at least 48 hours) at least twice in the ICU, as recommended by the author of FS-ICU 24R^(5,14)^. Thirty family members participated in this stage, selected by convenience, as suggested by the theoretical framework used^(16,21-23)^. Data collection with families took place during the New Coronavirus (SARS-CoV-2) pandemic in June 2021; therefore, with an approach restricted to telephone and online calls.

Family members’ contacts were obtained after the consent of the participating institution and from a spreadsheet with the list of patients admitted to the ICU of a teaching hospital in the first half of 2020. Of the 188 patients admitted during this period, 108 met the inclusion criteria and only 22 family members agreed to participate. Therefore, they were asked to indicate other family members to complete the 30. Family members were informed about the study’s objective, and a link with the Informed Consent Form was sent for online agreement.

Upon acceptance, the interviews were scheduled and recorded using a voice recorder and/or a voice recording application on the computer for later transcription. The interview was organized into four stages: 1) completion of a complementary characterization form to that provided by the hospital to better characterize patients; 2) before reading the items, participants were encouraged to discuss their experience of having a family member in the ICU - the decision to start with this question was intended to bring family members closer to this experience, helping them to recall the facts of hospitalization and minimize memory bias, since hospitalization had occurred more than a year ago; 3) PCV3 items were read without explanations up to three times, and if there was no understanding, it was recorded as a lack of response, justifying the difficulty presented; and 4) to assess the understanding of items, at the end of the interview, a questionnaire for general semantic analysis was applied.

#### Stage 7 - Sending the original version to the author

All reports and considerations from pre-test participants were stored in a Microsoft Word^®^ spreadsheet. From the recording of interviews, it was possible to transcribe the observations indicated in each item of the questionnaire, which underwent a thorough assessment, with double checking and criticality in selecting only observations on the understanding of items before sending it to the author of the questionnaire (stage VII), who agreed with the suggestions sent and resulted in the final Portuguese consensus version (FPCV).

## RESULTS

In the translation stage of the 41 aspects assessed in the questionnaire (30 items, main title, titles of the two domains and eight subtitles), 38 (93%) presented some discrepancy between PV1 and PV2. In translation synthesis, the phrase “Family Satisfaction with Care in the Intensive Care Unit: FS-ICU 24R” was translated as “*Satisfação Familiar com os Cuidados da Unidade de Terapia Intensiva: FS-ICU24R*” and “*Satisfação da Família com o cuidado prestado na Unidade de Terapia Intensiva: SF-UTI 24R*”, and in consensus, “*Satisfação da Família com os Cuidados na Unidade de Terapia Intensiva: FS-ICU 24R*”. “Satisfaction with care” was translated as “*Satisfação com o cuidado*” and “*Satisfação com o atendimento*”, and by consensus, the first one prevailed. There were also differences in the use of pronouns: “How did we treat you?” translated to “*Como te tratamos?” and “Como nós tratamos você?*”, opting for the latter; “How satisfied are you with...” with the translations “*Qual é teu nível de satisfação com...*” and “*Qual seu grau de satisfação com a atmosfera*...”, and by consensus, the latter was chosen.

There was consensus regarding terms that favored understanding, such as “atmosphere (mood) of the ICU” “*ambiente (clima) da UTI*” and “*atmosfera (humor) na UTI*”, opting for the latter to avoid confusion with room temperature. The term “rounds” has no translation into Portuguese, so “participation in daily rounds” was translated as “*participação nas visitações diárias*” and “*participação da avaliação diária do seu familiar*”, opting for the first one.

There was a consensus in the translations not to include “family member”, so for “family member (the patient)”, “*familiar (paciente)*” was kept, as well as in “critically ill family member” by “*familiar gravemente enfermo*” instead of “*paciente crítico*”, as it was felt that the latter might not be adequately understood by family members with a lower level of education.

Some synonyms also diverged in the translation of “skill” to “*qualificação*” and “*habilidade*”, opting for the latter; in the translation of “level” to “*nível*” and “*grau*”, opting for the latter; in the translation of “concern and caring” by “*interesse e cuidado*” in both translations; in the translation of “understanding of information” by “*clareza das informações*” in both translations, rather than adhering to the literal translation “*compreensão*”.

In this process, the terms chosen were those considered most appropriate, based on the translators’ and researchers’ consideration, from a semantic and idiomatic point of view, consensually maintaining the terms considered usual in the context.

Once the Portuguese consensus version stage was completed, the content validity stage began, based on the assessment by the PCV1 expert committee. Of the seven experts who participated in the first stage, five (71%) held a master’s degree and two held a specialist degree (29%). Concerning professional categories, one psychologist, one physician, two physiotherapists and three nurses participated. The overall CVI for each equivalence assessed - idiomatic, cultural and conceptual - reached values above 0.80. The semantic assessment presented a lower assessment (0.77), with a lower average among evaluators in the following elements: items 1 (0.71%), 2 (0.75%), 3 (0.86%), 4 (0.82%), 5 (0.82%), 7 (0.71%), 8 (0.79%), 10 (0.71%), 11 (0.68%), 12 (0.71%), 13 (0.71%), 15 (0.82%), 18 (0.82%), 19 (0.71%) and title of the second domain (0.64%). However, the overall mean reached 0.86. Considering that 90% would be equivalent to 6.3 experts, the value of 86% was considered acceptable, which corresponds to the approval of six experts.

Since the consensus version with experts from the first phase was not synchronous, the need for a second one was identified in order to discuss aspects with equivalence lower than 0.80, since values above 80% are recommended as acceptable. The participation of one of the translators was essential to maintain equivalence, especially semantic equivalence, which received the lowest assessment. In this second phase, an online meeting was held, in which the researchers, two intensive care specialists and doctors in nursing with experience in validity studies participated, as well as one of the translators from stage 1 to review and ensure pertinent and equivalent changes to the original instrument. In this second round, there was consensus among experts to maintain the acronym “*FS-ICU 24R/Brasil*”.

In back-translation, in EV1 and EV2, it was identified that, of the 41 aspects assessed, five (12%) were identical, and the others presented at least one divergent word, either because they were synonyms or due to each professional’s translation characteristic. As an example, the subtitle “The ICU staff” was translated as “The ICU team”. The items with the expressions “how well”, “how included” and “how supported”, which would be translated as “*quão bem*”, “*quão incluído*” and “*quão suportável*”, respectively, in back-translation, presented a slight divergence, but the translators chose not to use “*quão*”, since it is not widely used in Portuguese. The consensus version in English was approved in the first assessment by the author of the original questionnaire and authorized for pre-testing.

In relation to the profile of the 30 family members participating in pre-test, they made an average of 13.1 visits (SD = 16.2). The age range was between 26 and 71 years, with an average of 44.4 years (SD = 12.2); 21 were female; 19 were children, in addition to three grandchildren, two siblings, a daughter-in-law, an aunt, a father, a niece, a grandmother, and a wife. Regarding having ever accompanied another family member to the ICU, 20 (67%) reported “yes”. Concerning living with patients, 19 (63%) responded “no” and, of these, 13 visited them more than once a week. Six (20%) participants lived outside the city where the hospital was located. As for education, four (13%) did not complete high school; seven (23%) completed high school; two (7%) completed vocational training; six (20%) held a bachelor’s degree; and 11 (37%) held a graduate degree.

Of the four questions for PCV3 assessment, all had more than 85% approval by family members (Table 1). Of the 30, four commented on items that did not represent the situations experienced during patients’ hospitalization, such as: participation in the decision-making process and sufficient time to make a decision (items 12 and 24); frequency of communication with nurses (item 8) - they claimed that communication was exclusively with physicians.

**Table 1:** Mean overall assessment of the questionnaire by pre-test participants, Pelotas, Rio Grande do Sul, Brazil, 2022 (N=30)

Portuguese version consensus 3 overall assessment	n	%
Overall, how do you rate the questionnaire?		
Good	28	93.3
Average	2	6.7
Poor	-	-
How do you consider the questions in the questionnaire?		
Easy	26	86.7
Fair	4	13.3
Difficult	-	-
Did you have any difficulty responding to the response scale?		
Yes	-	-
More or less	4	13.3
No	26	86.7
Do you consider the questions important for assessing family satisfaction?		
Yes	28	93.3
More or less	2	6.7
No	-	-

*n - sample; % - percentage.*

As a suggestion for improvement, two family members commented on the answer options for question 26, understanding option 3 as redundant (“*Senti que ele(a) estava bastante confortável*”), when compared with option 4 (“*Senti que ele(a) estava muito confortável*”), and suggested removing “*bastante*” from option 3. This suggestion was considered relevant and sent for a second assessment by the main author, who agreed. This adjustment did not invalidate participants’ understanding of the questionnaire; therefore, after adaptation stages, FPCV was obtained.

The average length of interviews ranged from 18 minutes to 1 hour and 10 minutes, with an average of 32 minutes, as some family members felt the need to provide details about their experiences related to hospitalization. Family members were asked to assess the questionnaire in general (Table 2) and to feel free to make suggestions for modifications, which were transcribed and assessed.

**Table 2:** Correlation between items (r) and Cronbach’s alpha if an item is excluded for items in the “satisfaction with care” domain (N=30), Pelotas, Rio Grande do Sul, Brazil, 2022

Items	r	α
*1. A preocupação e o cuidado da equipe da UTI? Considerando a cortesia, o respeito e a compaixão que seu familiar (paciente) recebeu.*	0.837	0.899
*2a. Como a equipe da UTI avaliou e tratou a dor do seu familiar.*	0.283	0.917
*2b. Como a equipe da UTI avaliou e tratou a falta de ar do seu familiar.*	0.292	0.916
*2c. Como a equipe da UTI avaliou e tratou a agitação do seu familiar.*	0.296	0.916
*3. Como foi o interesse demonstrado pela equipe da UTI por suas necessidades.*	0.790	0.901
*4. Como foi o apoio emocional oferecido pela equipe da UTI para você.*	0.523	0.909
*5. Como foi o trabalho em equipe de todo o pessoal da UTI que cuidou do seu familiar.*	0.818	0.900
*6. Como considera a cortesia, o respeito e a compaixão que você recebeu.*	0.836	0.899
*7. Como considera o cuidado dos(as) enfermeiros(as) em relação ao seu familiar.*	0.783	0.901
*8. Considerando o número de vezes que os(as) enfermeiros(as) informaram sobre a condição do seu familiar.*	0.540	0.909
*9. Como você considera o cuidado dos(as) médicos(as) em relação ao seu familiar.*	0.839	0.899
*10. Qual seu grau de satisfação com o ambiente (humor) na sala de espera da UTI?*	0.495	0.910
*11. Qual seu grau de satisfação com o ambiente (humor) na UTI?*	0.363	0.914
*12. Qual seu grau de satisfação com sua participação nas decisões diárias referentes ao familiar?*	0.654	0.905
*13. Qual seu grau de satisfação com sua participação no cuidado do familiar gravemente doente?*	0.468	0.911
*14. Algumas pessoas querem que todo o possível seja feito por seus problemas de saúde, enquanto outras não desejam que se faça tanto. Qual seu grau de satisfação com o NÍVEL ou a quantidade de cuidado que seu familiar recebeu na UTI?*	0.827	0.900

*r - correlation; α - Cronbach’s alpha.*

In assessing item reliability, items 28, 29 and 30 were not considered, as they were open-ended questions and underwent the same criteria for translation, adaptation and content validity. The questionnaire showed adequate internal consistency, with a Cronbach’s alpha coefficient (α) of 0.93.

To assess the correlation between items and Cronbach’s alpha, the “satisfaction with care”, “family satisfaction with decision-making around care of critically ill patients” and “process of making decisions” domains were analyzed. The individual analysis of components resulted in α = 0.89, 0.91 and 0.79, respectively. It is worth noting that, at this stage of the study, the same items presented in the original version of the questionnaire were considered; however, as the third domain, the “process of making decisions” domain was adopted, which makes up the second part of the original questionnaire.

The first domain, “satisfaction with care”, showed high agreement (α = 0.89). However, items 2a (r = 0.2832), 2b (r = 0.2926) and 2c (r = 0.2969) presented a correlation lower than 0.30, but, when eliminating them, no improvement in the questionnaire consistency was observed. All other items in this domain presented a correlation above 0.30 (r ≥ 0.30) (Table 2).

The second domain, “family satisfaction with decision-making around care of critically ill patients”, also presents high agreement (α = 0.91), and all items present r ≥ 0.30 (Table 3).

**Table 3:** Correlation between items (r) and Cronbach’s alpha if an item is excluded for items in the “family satisfaction with decision-making around care of critically ill patients” domain (N=30), Pelotas, Rio Grande do Sul, Brazil, 2022

Items	r	α
*15. Quanto ao número de vezes que os(as) médicos(as) comunicaram sobre a condição clínica do seu familiar.*	0.583	0.922
*16. Quanto a disponibilidade da equipe da UTI para responder às suas perguntas.*	0.756	0.898
*17. Quanto a clareza das informações transmitidas pela equipe da UTI.*	0.880	0.880
*18. Quanto a honestidade (transparência) das informações transmitidas sobre a condição do seu familiar.*	0.845	0.885
*19. Quanto às informações da equipe da UTI sobre o que estava acontecendo com seu familiar e o porquê alguns procedimentos foram realizados.*	0.842	0.886
*20. Quanto a consistência das informações fornecidas sobre a condição do seu familiar (se você recebeu informações semelhantes por parte do médico, enfermeiro, etc.).*	0.652	0.913

*r - correlation; α - Cronbach’s alpha.*

The “process of making decisions” domain also showed high agreement (α = 0.79), and the correlation of items with the total is satisfactory (r ≥ 0.30) (Table 4).

**Table 4:** Correlation between items (r) and Cronbach’s alpha if an item is excluded for items in the “process of making decisions” domain, Pelotas, Rio Grande do Sul, Brazil, 2022

Items	r	α
*21. Em que medida você se sentiu incluído(a) ou excluído(a) no processo de tomadas de decisão?*	0.6914	0.7393
*22. Em que medida você se sentiu apoiado(a) no processo de tomadas de decisão?*	0.6233	0.7526
*23. Você sentiu que teve controle sobre os cuidados ao seu familiar?*	0.5212	0.7719
*24. Ao tomar decisões, você teve tempo suficiente para que suas dúvidas fossem consideradas e suas perguntas respondidas?*	0.3712	0.7989
*25. Qual das seguintes opções melhor descreve sua opinião:*	0.3369	0.8048
*26. Durante as últimas horas de vida do seu familiar, qual das seguintes opções melhor descreve sua opinião:*	0.5815	0.7607
*27. Nas últimas horas antes do falecimento do seu familiar, qual das seguintes opções melhor descreve sua opinião:*	0.5830	0.7604

*r - correlation; α - Cronbach’s alpha.*

Considering the 29 items of the questionnaire, Cronbach’s alpha coefficient returned to the value of 0.93. When performing the correlations (r) between items and Cronbach’s alpha (if the item was excluded), items 2a, 2b and 2c continued to present correlation below r ≤ 0.30, being 0.2509, 0.2501 and 0.2634, respectively.

## DISCUSSION

FS-ICU 24R, reformulated in 2018, allows ICU professionals to evaluate care practices based on the assessment of family members’ satisfaction^(6)^. Although it presents discreet modifications when compared to FS-ICU 24, the authors recommend the use of the new version. However, after reviewing literature, there was no evidence of adaptation of the new version to other cultures, which limits the comparison of this discussion.

The use of the questionnaire in Brazil initially depends on the cross-cultural adaptation process, which met the objective of carrying out cross-cultural adaptation and validating the FS-ICU 24R content for use in Brazil, which demonstrated satisfactory equivalences and an adequate mean CVI in all items. This assessment becomes relevant to obtain the equivalences necessary for the questionnaire^(24)^ applicability and reproducibility which, in this case, is intended for Brazilian Portuguese.

Adapting instruments requires a rigorous and consistent methodology to ensure linguistic qualities between the original and translated versions^(25,26)^. During the translation stage, discrepancies related to the style of the two translators became evident: one presented a literal and informal version, and the other adapted it to better understand the Portuguese version. Therefore, semantic equivalence was sought in each item compared to the original version, in a meticulous process of comparison and modification, as recommended in classical literature^(16,21,22)^.

It has become clear that the adaptation process is extensive and laborious. It contains systematic stages that depend on collective dedication and, nevertheless, active participation by the authors. Above all, the translation process stands out, as it is not a simple translation and, evidently, it is from this that the following stages were developed.

Most studies that adapted FS-ICU 24 addressed the importance of translation and back-translation^(4,7-9,12,13)^. However, only in Brazil^(4)^, Germany^(7)^ and China^(9)^ did the authors detail the assessment of equivalences, with semantics only being mentioned in the study of adaptation of FS-ICU 24 version to Brazilian Portuguese^(4)^.

Despite the dissemination of psychometric studies, methodological rigor continues to be one of the pillars to be reinforced for its correct use^(27)^; however, homogeneity and clarity are still not perceived in this process. In relation to the specification of the literature chosen for the method, among the adaptation studies of FS-ICU 24, only in Brazil^(4)^ and Thailand^(12)^ did they mention meeting the requirements of authors of classical psychometrics^(16,21)^.

Regarding the order of stages, consolidated literature follows expert assessment after back-translation^(16,17)^, but this study was in line with the proposal of previous Brazilian publications^(4,18,19)^, maintaining the back-translation stage after the expert committee, which makes it possible to identify errors of meaning before back-translation.

Regarding the main author’s authorization, contact was made via email, as occurred in the adaptations carried out in other countries such as Brazil^(4)^, South Korea^(8)^, China^(9)^, Norway^(11)^, Thailand^(12)^ and Turkey^(13)^, in line with the literature^(22)^, which recommends authorization from the main author of the original questionnaire to carry out adaptation and validity.

In terms of content validity, equivalence was found between the original questionnaire and the Portuguese version, with an overall average of 0.86 in semantic, idiomatic, cultural and conceptual equivalences, which is equivalent to 80% acceptance as recommended^(23)^.

In this scenario, content validity proved to be reliable because it included a diversity of professionals, with different perceptions and actions with critical patients and their families. It is worth noting that, for assertive decision-making, specialists invited to the assessment stage must have training, qualifications and experience in the area of the questionnaire assessed, since they will be jointly responsible for the pre-final version^(24)^.

To identify inconsistencies or weaknesses in the translation process, a semantic assessment with the population for which the instrument is intended is recommended^(19)^. Priority was given to semantic assessment at different levels of education as recommended^(27)^ to obtain understanding of the item by the target population with a lower level of education. It is assumed that the absence of difficulties in understanding the terms is related to the fact that most respondents have a high level of education (undergraduate/graduate degrees).

The results obtained in pre-test ensure the FS-ICU24R adequate adaptability to Brazilian Portuguese, as occurred in adaptations of FS-ICU 24 in Brazil^(4)^, Germany^(7)^, South Korea^(8)^, China^(9)^, Iran^(10)^, Norway^(11)^, Thailand^(12)^ and Turkey^(13)^. It is noteworthy that, in the studies in the United Kingdom, cross-cultural adaptations were not carried out, since the native language of the questionnaire is English, and it was only reviewed by the target population and healthcare professionals^(28,29)^.

Cronbach’s alpha of FS-ICU 24R in the questionnaire subscales was above 0.70 (α=0.89, α=0.91 and α=0.79), demonstrating high reliability in pre-test. However, the correlation of items 2a, 2b and 2c was less than 0.3. Thus, according to pre-test results, these items needed to be removed; however, it was decided to maintain them until FS-ICU 24R Brazil is applied to a larger sample, as in the validity test.

When considering the 29 items of the questionnaire, Cronbach’s alpha coefficient presented a value of 0.93, which represents a high value. The number of items affects the instrument consistency^(30)^. Thus, questionnaires with many items increase Cronbach’s alpha value, and this does not necessarily imply an increase in the questionnaire internal consistency; on the contrary, a low alpha value may simply mean that there are few items.

Strategies used in this study, such as organization, process detailing and researcher involvement in the different stages, allow the identification of “problem” items, which are difficult to reach consensus on in translation, with less assessment of equivalences and, consequently, reflected in statistical analysis with lower correlations (r) between items, as was the case with items 2a, 2b and 2c. Thus, the well-presented method provides support for the researcher to observe each stage, in order to obtain clarity and identify the weaknesses of items.

Of the studies that adapted FS-ICU 24, those that did not demonstrate a preliminary assessment of the psychometric properties prevailed^(4,8,9,11-13)^, which makes comparisons between the results impossible. In this study, pre-test was performed to verify the understanding of items in a pilot sample. Therefore, caution must be exercised before drawing hasty conclusions based on preliminary data.

In Brazil, the restrictive visitation model still prevails^(31)^, similar to a Latin American study, with a maximum of six hours per day of family involvement in visits^(32)^. This restriction of institutions on co-participation, based on non-adherence to extended visitation, contributes to the family member’s perception of not belonging, as observed in items about participation in the decision-making process and sufficient time to make a decision, which did not represent situations experienced by some family members in this adaptation.

In Chinese culture, for instance, the family plays a fundamental role in decision-making, where respect for older adults predominates, with values consistent with family responsibility and harmonious human relationships^(9)^. The process of adapting FS-ICU 24R in Brazil and subsequently validating it could be a precursor to comparing and analyzing cultural variability, especially regarding family members’ expectations regarding the decision-making process and communication with the ICU team.

### Study limitations

As a study limitation, it is worth noting that pre-test was carried out with a sample selected for convenience and that most of family members in the sample had at least completed high school, which can be justified by online data collection, which could distance those with a lower level of education. Therefore, the aim is to minimize this limitation in assessment of its psychometric properties, since the sample will be larger. It is worth considering that responses were self-reported, and this may imply response and memory bias, since collection was carried out more than a year after hospitalization. Therefore, it is possible that, when applying this questionnaire to other populations of family members, the same results will not be observed.

### Contributions to nursing

The research contributes to the possibility of assessing the satisfaction of family members of ICU patients through the use of a questionnaire adapted to Brazilian Portuguese, since it achieved adequate reliability in the proposed cross-cultural adaptation process. However, it is still necessary to verify the psychometric properties of FS-ICU 24R/Brazil in order to guarantee its validity and reliability.

## CONCLUSIONS

The development of FS-ICU 24R version adapted to Brazilian Portuguese proved to be easy to apply and understand. It met equivalence criteria between the original and translated questionnaires, and its content was validated by experts. The pre-test showed that the questionnaire presents internal consistency of items and, therefore, is capable of assessing the satisfaction of families of ICU patients.

Thus, it is concluded that FS-ICU 24R/Brazil is adequate for testing its psychometric properties, in order to guarantee validity and reliability, which may contribute to strengthening research in institutions engaged in involving the family in the hospital admission process, especially in developing strategies that ensure family satisfaction maintenance during the hospitalization of critically ill patients in the ICU.

The follow-up of this study, with FS-ICU 24R/Brazil validity, will allow the comparison of results with different realities, since the previous versions of this instrument have already been validated in several countries, such as Chile, Spain and China, and have contributed to a reflection on family participation in care and decision-making in these services, favoring quality of care. Thus, with the availability of FS-ICU 24R, it is expected to identify aspects that need to be improved and those that aroused family satisfaction and deserve to be maintained and strengthened, in addition to contributing to the promotion of strategies that drive research on family involvement in co-participation in care and decisions.

## Data Availability

The research data are available within the article.
