# Supplementary material for: Cross-cultural adaptation of Family Satisfaction with Care in the Intensive Care Unit 24R - Brazilian version
Source: Rev Bras Enferm. 2025 Dec 12;78(Suppl 4):e20240273. doi: 10.1590/0034-7167-2024-0273 (PMC12707774; doi:10.1590/0034-7167-2024-0273)
Supplement: Translation

# Translation: Adaptação transcultural do *Family Satisfaction with Care in the Intensive Care Unit* 24R - versão brasileira

**DOI:** 10.1590/0034-7167-2024-0273pt

**Authors:** Josiele de Lima Neves, Eda Schwartz, Lílian Moura de Lima Spagnolo, Eraldo Schunk Silva, Andrieli Daiane Zdanski de Souza, Fernanda Lise

**Affiliations:** I Universidade Federal de Pelotas. Pelotas, Rio Grande do Sul, Brasil; II Hospital de Clínicas de Porto Alegre. Porto Alegre, Rio Grande do Sul, Brasil; III Fundação Universidade Rio Grande. Rio Grande, Rio Grande do Sul, Brasil; IV Universidade Estadual de Maringá. Maringá, Paraná, Brasil

**Keywords:** Família, Satisfação, Unidades de Terapia Intensiva, Estudo de Validação, Enfermagem.

## Abstract

**Objetivos:**

realizar adaptação transcultural e validar o conteúdo do *Family Satisfaction with Care in the Intensive Care Unit* 24R para uso no Brasil.

**Métodos:**

pesquisa metodológica. Realizaram-se tradução, consenso da tradução, avaliação das equivalências, retrotradução, consenso da retrotradução, pré-teste. Sete especialistas avaliaram equivalências da tradução e validade de conteúdo. Aplicabilidade, compreensão dos itens e consistência interna foram verificadas em pré-teste com 30 familiares. Os dados foram analisados com o Índice de Validade de Conteúdo, avaliação semântica, correlação (r) entre itens e alfa de Cronbach.

**Resultados:**

ajustes linguísticos foram realizados para tornar os itens compreensíveis. O Índice de Validade de Conteúdo foi de 0,86, o alfa de Cronbach, 0,93, e todos os itens apresentaram correlação ≥0,30. Avaliação semântica obteve mais de 85% de aprovação.

**Conclusões:**

o questionário adaptado teve seu conteúdo validado, demonstrando ser confiável para avaliação das propriedades psicométricas. Apresentou potencial para contribuir na avaliação da satisfação familiar na terapia intensiva no Brasil, a fim de identificar aspectos que precisam ser melhorados, assim como os que devem ser mantidos e fortalecidos.

## INTRODUÇÃO

A admissão de pacientes na Unidade de Terapia Intensiva (UTI) geralmente inclui o envolvimento de familiares próximos. Ademais, considera-se o termo “familiares” pessoas intimamente envolvidas no processo de admissão e permanência do paciente na UTI, como cônjuge, filhos ou pais, mas também amigos e pessoas próximas ao paciente^(1)^. Para melhorar a qualidade do cuidado prestado às famílias, é importante avaliar a satisfação dos familiares com os cuidados e o apoio que receberam para identificar áreas que necessitam ser melhoradas^(2)^.

Nessa conjuntura, para avaliar a satisfação do familiar, faz-se necessário a utilização de instrumentos confiáveis, validados e com abrangente utilização, os quais podem contribuir para a gestão do cuidado em saúde. Neste sentido, a Política Nacional de Humanização da Atenção e Gestão em Saúde no SUS, conhecida como HumanizaSUS, contribui para a melhoria da gestão do cuidado, já que, sendo inclusiva e resolutiva, oferece inovações nas práticas gerenciais, fortalecendo a relação entre paciente, equipe e família^(3)^.

Apesar de a avaliação da satisfação da família com cuidados, ambiente, equipe e tomada de decisão ser uma temática incipiente na realidade brasileira, evidencia-se progresso na produção científica. Em 2018, no Brasil, foi apresentada uma versão confiável para o português brasileiro^(4)^ do questionário canadense *Family Satisfaction with Care in the Intensive Care Unit* (FS-ICU 24)^(5)^. No mesmo ano, os autores do instrumento original disponibilizaram a versão revisada do FS-ICU 24R, na qual foram realizadas modificações de alguns itens e recebeu o acréscimo da letra “R”, de *revised*, após a sigla^(6)^.

Na literatura, identifica-se que o FS-ICU 24^(5)^ é reconhecido por apoiar a equipe e família na tomada de decisão e apresenta ampla utilização em estudos internacionais, tendo sido adaptado nos países como Brasil^(4)^, Alemanha^(7)^, Coréia do Sul^(8)^, China^(9)^, Irã^(10)^, Noruega^(11)^, Tailândia^(12)^ e Turquia^(13)^. Identificou-se a necessidade de realizar a validação do conteúdo e semântica da nova versão do FS-ICU 24R para tornar os itens compreensíveis e adequados, permitindo proceder com segurança para o teste de validação do instrumento no contexto brasileiro e, assim, contribuir para avanços na gestão do cuidado, oferecendo uma versão atualizada, confiável e válida para ser testada com famílias brasileiras em UTIs.

## OBJETIVOS

Realizar adaptação transcultural e validar o conteúdo do FS-ICU 24 para uso no Brasil.

## MÉTODOS

### Aspectos éticos

Antes da realização da pesquisa, foi concedida autorização, com comunicação via correio eletrônico, pelo autor que detém os direitos do questionário. Todos os procedimentos realizados respeitaram os princípios éticos, sendo a pesquisa aprovada pelo Comitê de Ética em Pesquisa.

### Delineamento do estudo

Trata-se de estudo metodológico de adaptação transcultural da versão revisada^(6)^ do FS-ICU 24^(5)^ do inglês para o português do Brasil, desenvolvido entre fevereiro de 2019 e setembro de 2021. Para seu desenvolvimento, seguiu-se o *checklist* para estudos qualitativos.

O FS-ICU 24R é composto por 24 itens que permitem avaliar a satisfação da família com os cuidados, o ambiente, a equipe e a sua participação na tomada de decisão^(6)^. Entre eles, o item 2 é subdividido em 2a, 2b e 2c, o que operacionalmente acrescenta dois itens. Além disso, o questionário contempla a avaliação dos familiares daqueles que faleceram, através dos itens 25, 26 e 27. Diante disso, operacionalmente serão avaliados 29 itens. Originalmente, os itens estão distribuídos em dois domínios: satisfação com o cuidado; e satisfação da família com a tomada de decisões em relação ao cuidado com os pacientes graves^(6,14)^.

As opções de respostas para os itens 1 até 20 são apresentadas em uma escala do tipo Likert de cinco pontos que varia de 1 (muito insatisfeito) a 5 (completamente satisfeito), acrescida da opção não se aplica (N/A). Entre os itens 21 e 27, as alternativas de resposta são organizadas em conjuntos de cinco ideias para a escolha daquela que expresse melhor a opinião. Para ambos, quanto maior a pontuação obtida, mais favorável é a satisfação do familiar^(14)^.

A confiabilidade do questionário foi verificada pela consistência interna, mediante aplicação do coeficiente alfa de Cronbach, testando-se a mudança no seu valor conforme a retirada hipotética de itens, com verificação das correlações entre as respostas. Consideraram-se como ideal valores entre 0,7 e 0,9, e na análise da correlação dos itens, foram considerados aptos os itens com correlação acima de 0,3^(15)^.

O processo de tradução e adaptação foi baseado nas recomendações do *Guidelines for the Process of Cross-Cultural Adaptation of Self Report Measures* ^(16)^, com alteração na ordem das etapas^(17)^ e em consonância com estudos brasileiros^(18,19)^. Compreendeu as seguintes etapas (Figura 1): I tradução para o português do Brasil; II consenso das versões em português; III avaliação por um comitê de especialistas; IV retrotradução para o idioma original; V consenso das versões em inglês e comparação com a versão original; VI pré-teste; e VII envio ao autor da versão original.

**Figura 1:** Fluxograma da trajetória metodológica de adaptação transcultural do *Family Satisfaction with Care in the Intensive Care Unit* 24R para uso no Brasil, Pelotas, Rio Grande do Sul, Brasil, 2022

*VOI - versão original em inglês; VP1 - versão em português tradutor 1; VP2 - versão em português tradutor 2; VPC1 - versão em português consenso 1; VPC2 - versão em português consenso 2; VI1 - versão em inglês tradutor 1; VI2 - versão em inglês tradutor 2; VIC - versão em inglês consenso; VPC3 - versão português consenso 3; VF - versão final.*

### Estudo de campo

#### Etapas 1 e 2 - Traduções e consenso das versões em português

A etapa I, que corresponde à tradução para o português do Brasil, foi realizada de forma independente por duas tradutoras brasileiras (tradutora 1 e tradutora 2), as quais receberam uma carta-convite com solicitação de uma tradução que preservasse a equivalência semântica dos itens originais. Os critérios de inclusão foram ser brasileiro, ter domínio da língua inglesa e amplo conhecimento da cultura canadense. Cada tradutora produziu um atestado de tradução e um relatório das versões em português. Após a obtenção das duas versões, versão em português 1 (VP1) e versão em português 2 (VP2), foi realizada a etapa II, em que as duas tradutoras estabeleceram contato em reunião para discussão e síntese das versões traduzidas, surgindo, então, a versão em português consenso 1 (VPC1).

#### Etapa 3 - Análise pelo comitê de especialistas

Na etapa III, foi realizada a análise por um comitê composto por setes especialistas. Os membros deste comitê foram convidados a partir de alguns critérios, os quais todos atenderam: ser profissional da área da saúde, experiente com o método desta pesquisa e/ou com o idioma inglês e/ou com a terapia intensiva. Esta etapa foi desenvolvida em dois momentos: 1º) com os sete especialistas, verificaram-se as equivalências semântica, idiomática, cultural e conceitual entre a versão traduzida e sintetizada (VPC1) e comparada com a versão original. Assim, a proporção ou porcentagem de concordância entre os especialistas foi verificada por meio do Índice de Validade de Conteúdo (IVC). A adequação de cada item variou entre -1 (não equivalente), 0 (não é possível avaliar/não sei) e +1 (equivalente), conforme estudo semelhante^(20)^. No segundo momento, para garantir compreensão e fidedignidade da versão consenso, os itens e domínios com menor percentual de equivalências foram reavaliados, simultaneamente, em reunião virtual por uma tradutora da primeira etapa, duas enfermeiras especialistas em terapia intensiva e com *expertise* em adaptação e validação de instrumentos, e as pesquisadoras como mediadoras. Após ajustes, a versão em português consenso 2 (VPC2) foi submetida à retrotradução.

#### Etapa 4 - Retrotradução para o idioma original

Na etapa IV, retrotradução, dois tradutores canadenses (tradutor 3 e tradutor 4), com amplo domínio do português do Brasil, conhecedores da cultura brasileira e residentes no Brasil, foram convidados à tradução da VPC2, gerando duas retrotraduções independentes, denominadas versões em inglês 1 e 2 (VI1 e VI2).

#### Etapa 5 - Consenso das versões em inglês e comparação com a versão original

A etapa V consiste em garantir que a versão traduzida esteja refletindo o mesmo conteúdo que a versão original. Organizou-se uma reunião virtual com o objetivo de avaliar as equivalências entre o questionário original e as retrotraduções, com a participação de uma das tradutoras da retrotradução, uma enfermeira com vasto conhecimento do idioma inglês e de estudos metodológicos, mediada pelas pesquisadoras. Após a reunião, a versão em inglês consenso 1 (VIC1) foi enviada para revisão do autor do questionário. Após ajustes, a versão foi traduzida ao português e denominada versão em português consenso 3 (VPC3).

#### Etapa 6 - Pré-teste com a população-alvo

Na etapa VI, foi realizado o pré-teste, que consistiu na aplicação da VPC3 à população-alvo, que atendeu aos seguintes critérios: ter mais de 18 anos, ter fluência na língua portuguesa do Brasil, ter visitado o paciente (internado por pelo menos 48 horas) no mínimo duas vezes na UTI, conforme recomendação do autor do FS-ICU 24R^(5,14)^. Participaram desta etapa 30 familiares, selecionados por conveniência, conforme sugere o referencial teórico utilizado^(16,21-23)^. A coleta de dados com as famílias ocorreu durante a pandemia do Novo Coronavírus (SARS-CoV-2) em junho de 2021; portanto, com abordagem restrita a ligação telefônica e *online*.

Os contatos dos familiares foram obtidos após anuência da instituição participante e a partir de uma planilha com a relação dos pacientes internados na UTI, de um hospital escola, no primeiro semestre de 2020. Dos 188 pacientes internados neste período, 108 atenderam aos critérios de inclusão e apenas 22 familiares aceitaram participar. Diante disso, foi solicitada indicação de outros familiares para completar os 30. Os familiares foram informados sobre o objetivo do estudo, sendo enviado um *link* com o Termo de Consentimento Livre e Esclarecido para concordância *online*.

Após o aceite, as entrevistas foram agendadas e gravadas por gravador de voz e/ou por aplicativo de gravação de voz no computador para posterior transcrição. A entrevista foi organizada em quatro momento: 1º) preenchimento de um formulário de caracterização complementar àquele disponibilizado pelo hospital para melhor caracterizar o paciente; 2º) antes da leitura dos itens, os participantes foram instigados a discorrer sobre como foi a experiência de ter um membro da família na UTI - a decisão de iniciar com esta pergunta teve o intuito de aproximar o familiar dessa vivência, auxiliando-o a rememorar os fatos da internação e minimizar o viés de memória, já que a internação ocorrera há mais de um ano; 3º) os itens da VPC3 foram lidos sem explicações por até três vezes, e caso não houvesse compreensão, era registrado como ausência de resposta, justificando qual a dificuldade apresentada; e 4º) para avaliar a compreensão dos itens, no final da entrevista, foi aplicado um questionário para análise semântica geral.

#### Etapa 7 - Envio ao autor da versão original

Todos os relatórios e considerações dos participantes do pré-teste foram armazenados em uma planilha do *software Microsoft Word^®^*. A partir da gravação das entrevistas, foi possível a transcrição das observações apontadas em cada item do questionário, as quais passaram por avaliação minuciosa, com dupla checagem e criticidade em selecionar apenas observações sobre o entendimento do item antes do envio ao autor do questionário (etapa VII), que concordou com as sugestões enviadas e resultou na versão em português consenso final (VPCF).

## RESULTADOS

Na etapa de tradução dos 41 aspectos avaliados no questionário (30 itens, título principal, títulos dos dois domínios e oito subtítulos), 38 (93%) apresentaram alguma discrepância entre a VP1 e VP2. Na síntese das traduções, a frase “*Family Satisfaction with Care in the Intensive Care Unit:* FS-ICU 24R” foi traduzida como “Satisfação Familiar com os Cuidados da Unidade de Terapia Intensiva: FS-ICU24R” e “Satisfação da Família com o cuidado prestado na Unidade de Terapia Intensiva: SF-UTI 24R”, e em consenso, “Satisfação da Família com os Cuidados na Unidade de Terapia Intensiva: FS-ICU 24R”. “*Satisfaction with care*” foi traduzido como “Satisfação com o cuidado” e “Satisfação com o atendimento”, e em consenso, prevaleceu a permanência da primeira. Houve diferenças também em uso de pronomes: “*How did we treat you*?” traduzido para “Como te tratamos?” e “Como nós tratamos você?”, optando-se pelo último; “*How satisfied are you with*...” com as traduções “Qual é teu nível de satisfação com...” e “Qual seu grau de satisfação com a atmosfera...”, e em consenso, optou-se pela última.

Houve consenso em relação a termos que favorecessem a compreensão, como “*atmosphere* (*mood*) *of the ICU*” por “ambiente (clima) da UTI” e “atmosfera (humor) na UTI”, optando-se pelo último para evitar confusão com a temperatura do ambiente. O termo “*rounds*” não possui tradução para o português, então “*participation in daily rounds*” foi traduzido como “participação nas visitações diárias” e “participação da avaliação diária do seu familiar”, optando-se pela primeira.

Foi consenso nas traduções a não inclusão de “membro da família”, então, para “*family member* (*the patient*)”, manteve-se “familiar (paciente)”, assim como em “*critically ill family member*” por “familiar gravemente enfermo” ao invés de “paciente crítico”, pois julgou-se que a última poderia não ser adequadamente compreendida pelos familiares de menor nível de escolaridade.

Alguns sinônimos também divergiram na tradução de “*skill*” para “qualificação” e “habilidade”, optando-se pelo último; na tradução de “*level*” para “nível” e “grau”, optando-se pelo último; na tradução de “*concern and caring*” por “interesse e cuidado” nas duas traduções; na tradução de “*understanding of information*” por “clareza das informações” nas duas traduções, ao invés de aderir à tradução literal “compreensão”.

Nesse processo, os termos escolhidos foram os considerados mais adequados, a partir da consideração dos tradutores e pesquisadores, partindo do ponto de vista semântico e idiomático, mantidos de forma consensual os termos considerados usuais ao contexto.

Finalizada a etapa consensual das versões em português, iniciou-se a de validação de conteúdo, a partir da avaliação pelo comitê de especialistas da VPC1. Dos sete especialistas que participaram da primeira fase, cinco (71%) possuíam título de mestre e, dois, de especialista (29%). Quanto às categorias profissionais, participaram um psicólogo, um médico, dois fisioterapeutas e três enfermeiros. O IVC geral para cada equivalência avaliada - idiomática, cultural e conceitual - atingiu valores acima de 0,80. Já a avaliação semântica apresentou menor avaliação (0,77), com menor média entre os avaliadores nos seguintes elementos: itens 1 (0,71%), 2 (0,75%), 3 (0,86%), 4 (0,82%), 5 (0,82%), 7 (0,71%), 8 (0,79%), 10 (0,71%), 11 (0,68%), 12 (0,71%), 13 (0,71%), 15 (0,82%), 18 (0,82%), 19 (0,71%) e título do 2º domínio (0,64%). No entanto, a média geral atingiu 0,86. Considerando que 90% equivaleriam a 6,3 especialistas, foi considerado o valor de 86% como aceitável, o que corresponde à aprovação de seis especialistas.

Em virtude da versão consenso com os especialistas da primeira fase não ser síncrona, identificou-se a necessidade de uma segunda, a fim de discutir aspectos com equivalência menor de 0,80, já que se recomendam valores acima de 80% como aceitáveis. A participação de uma das tradutoras foi fundamental para manter a equivalência, principalmente a semântica, que obteve a menor avaliação. Nesta segunda fase, realizou-se uma reunião *online*, em que participaram as pesquisadoras, duas especialistas em terapia intensiva e doutoras em enfermagem com experiência em estudos de validação, uma das tradutoras da etapa 1 para revisar e garantir alterações pertinentes e equivalentes ao instrumento original. Nesta segunda rodada, houve consenso entre os especialistas por manter a sigla “FS-ICU 24R/Brasil”.

Na retrotradução, nas versões VI1 e VI2, identificou-se que, dos 41 aspectos avaliados, cinco (12%) foram idênticos, e os demais apresentaram ao menos uma palavra divergente, seja por serem sinônimas ou pela característica de tradução de cada profissional. Como exemplo, o subtítulo “*The ICU staff*” foi traduzido por “*The ICU team*”. Os itens com a expressão “*how well*”, “*how included*” e “*how supported*”, que seriam traduzidos por “quão bem”, “quão incluído” e “quão suportável”, respectivamente, na retrotradução, apresentaram discreta divergência, mas os tradutores optaram por não utilizar “quão”, já que não representa ampla utilização no português. A versão consenso em inglês foi aprovada na primeira avaliação do autor do questionário original e autorizada para o pré-teste.

Em relação ao perfil dos 30 familiares participantes do pré-teste, realizaram em média 13,1 visitas (DP= 16,2). A variabilidade de idade foi entre 26 e 71 anos, com média de 44,4 anos (DP = 12,2); 21 eram do sexo feminino; 19 eram filhos, além de três netos, dois irmãos, uma nora, uma tia, um pai, uma sobrinha, uma avó, uma esposa. Sobre já terem acompanhado outro familiar na UTI, 20 (67%) relataram que “sim”. Em relação a morar com o paciente, 19 (63%) responderam que “não” e, desses, 13 os visitavam mais de uma vez na semana. Seis (20%) participantes residiam fora da cidade onde o hospital estava localizado. No que diz respeito à escolaridade, quatro (13%) não concluíram o ensino médio; sete (23%) possuíam ensino médio; dois (7%) possuíam ensino técnico; seis (20%) possuíam graduação; e 11 (37%) possuíam pós-graduação.

Das quatro questões para avaliação da VPC3, todas apresentaram mais de 85% de aprovação pelos familiares (Tabela 1). Dos 30, quatro comentaram sobre itens que não representavam as situações vivenciadas durante a internação do paciente como: participação no processo de tomada de decisão e tempo suficiente para tomar decisão (itens 12 e 24); frequência de comunicação com os enfermeiros (item 8) - alegaram que a comunicação era exclusivamente com os médicos.

**Tabela 1:** Média da avaliação geral do questionário pelos participantes do pré-teste, Pelotas, Rio Grande do Sul, Brasil, 2022 (N=30)

Avaliação geral da versão em português consenso 3	n	%
De modo geral, como você avalia o questionário?		
Bom	28	93.3
Regular	2	6.7
Ruim	-	-
Como você considera as questões do questionário?		
Fáceis	26	86.7
Regular	4	13.3
Difíceis	-	-
Você teve alguma dificuldade em responder à escala de respostas?		
Sim	-	-
Mais ou menos	4	13.3
Não	26	86.7
Você considera as perguntas importantes para avaliação da satisfação da família?		
Sim	28	93.3
Mais ou menos	2	6.7
Não	-	-

*n - amostra; % - porcentagem.*

Como sugestão de melhoria, dois familiares comentaram sobre as opções de resposta da questão 26, entendendo como redundante a opção 3 (“Senti que ele(a) estava bastante confortável”), ao ser comparada com a opção 4 (“Senti que ele(a) estava muito confortável”), e sugeriram a retirada do “bastante” da opção 3. Esta sugestão foi considerada relevante e enviada para segunda avaliação do autor principal, que concordou. Este ajuste não invalidou a compreensão do questionário pelos participantes; logo, após as etapas de adaptação, obteve-se a VPCF.

O tempo médio das entrevistas variou entre 18 minutos e 1 hora e 10 minutos, com média de 32 minutos, pois alguns familiares sentiram necessidade de detalhar sobre as experiências relacionadas à internação. Foi solicitado aos familiares que avaliassem o questionário de maneira geral (Tabela 2), além de ficarem à vontade para apresentar sugestões de modificações, as quais foram transcritas e avaliadas.

**Tabela 2:** Correlação entre itens (r) e alfa de Cronbach se o item for excluído para os itens do domínio “satisfação com o cuidado” (N=30), Pelotas, Rio Grande do Sul, Brasil, 2022

Itens	r	α
1. A preocupação e o cuidado da equipe da UTI? Considerando a cortesia, o respeito e a compaixão que seu familiar (paciente) recebeu.	0,837	0,899
2a. Como a equipe da UTI avaliou e tratou a dor do seu familiar.	0,283	0,917
2b. Como a equipe da UTI avaliou e tratou a falta de ar do seu familiar.	0,292	0,916
2c. Como a equipe da UTI avaliou e tratou a agitação do seu familiar.	0,296	0,916
3. Como foi o interesse demonstrado pela equipe da UTI por suas necessidades.	0,790	0,901
4. Como foi o apoio emocional oferecido pela equipe da UTI para você.	0,523	0,909
5. Como foi o trabalho em equipe de todo o pessoal da UTI que cuidou do seu familiar.	0,818	0,900
6. Como considera a cortesia, o respeito e a compaixão que você recebeu.	0,836	0,899
7. Como considera o cuidado dos(as) enfermeiros(as) em relação ao seu familiar.	0,783	0,901
8. Considerando o número de vezes que os(as) enfermeiros(as) informaram sobre a condição do seu familiar.	0,540	0,909
9. Como você considera o cuidado dos(as) médicos(as) em relação ao seu familiar.	0,839	0,899
10. Qual seu grau de satisfação com o ambiente (humor) na sala de espera da UTI?	0,495	0,910
11. Qual seu grau de satisfação com o ambiente (humor) na UTI?	0,363	0,914
12. Qual seu grau de satisfação com sua participação nas decisões diárias referentes ao familiar?	0,654	0,905
13. Qual seu grau de satisfação com sua participação no cuidado do familiar gravemente doente?	0,468	0,911
14. Algumas pessoas querem que todo o possível seja feito por seus problemas de saúde, enquanto outras não desejam que se faça tanto. Qual seu grau de satisfação com o NÍVEL ou a quantidade de cuidado que seu familiar recebeu na UTI?	0,827	0,900

*r - correlação; α - alfa de Cronbach.*

Na avaliação da confiabilidade dos itens não foram considerados, os 28, 29 e 30, por serem perguntas abertas, passaram pelos mesmos critérios de tradução, adaptação e validade de conteúdo. O questionário mostrou consistência interna adequada, com coeficiente alfa de Cronbach (α) de 0,93.

Para avaliação da correlação entre os itens e o alfa de Cronbach, foram analisados os domínios “satisfação com o cuidado”, “satisfação da família com a tomada de decisões em relação ao cuidado com os pacientes graves” e “processo de tomada de decisões”. A análise individual dos componentes resultou em α = 0,89, 0,91 e 0,79, respectivamente. Ressalta-se que, nesta etapa do estudo, foram considerados os mesmos itens apresentados na versão original do questionário; contudo, como terceiro domínio, adotou-se o domínio “processo de tomada de decisões”, que compõe a segunda parte do questionário original.

O primeiro domínio, “satisfação com o cuidado”, apresentou concordância alta (α= 0,89). No entanto, os itens 2a (r= 0,2832), 2b (r= 0,2926) e 2c (r= 0,2969) apresentam correlação menor a 0,30, mas, ao eliminá-los, não se observou melhoria na consistência do questionário. Todos os demais itens deste domínio apresentam correlação acima de 0,30 (r ≥ 0,30) (Tabela 2).

O segundo domínio, “satisfação da família com a tomada de decisões em relação ao cuidado com os pacientes graves”, também apresenta concordância alta (α= 0,91), e todos os itens apresentam r ≥ 0,30 (Tabela 3).

**Tabela 3:** Correlação entre itens (r) e alfa de Cronbach se o item for excluído para os itens do domínio “satisfação da família com a tomada de decisões em relação aos pacientes graves” (N=30), Pelotas, Rio Grande do Sul, Brasil, 2022

Itens	r	α
15. Quanto ao número de vezes que os(as) médicos(as) comunicaram sobre a condição clínica do seu familiar.	0,583	0,922
16. Quanto a disponibilidade da equipe da UTI para responder às suas perguntas.	0,756	0,898
17. Quanto a clareza das informações transmitidas pela equipe da UTI.	0,880	0,880
18. Quanto a honestidade (transparência) das informações transmitidas sobre a condição do seu familiar.	0,845	0,885
19. Quanto às informações da equipe da UTI sobre o que estava acontecendo com seu familiar e o porquê alguns procedimentos foram realizados.	0,842	0,886
20. Quanto a consistência das informações fornecidas sobre a condição do seu familiar (se você recebeu informações semelhantes por parte do médico, enfermeiro, etc.).	0,652	0,913

*r - correlação; α - alfa de Cronbach.*

O domínio “processo de tomada de decisões” também apresentou concordância alta (α= 0,79), e a correlação dos itens com o total é satisfatória (r ≥ 0,30) (Tabela 4).

**Tabela 4:** Correlação entre itens (r) e alfa de Cronbach se o item for excluído para os itens do domínio “processo de tomada de decisões”, Pelotas, Rio Grande do Sul, Brasil, 2022

Itens	r	α
21. Em que medida você se sentiu incluído(a) ou excluído(a) no processo de tomadas de decisão?	0,6914	0,7393
22. Em que medida você se sentiu apoiado(a) no processo de tomadas de decisão?	0,6233	0,7526
23. Você sentiu que teve controle sobre os cuidados ao seu familiar?	0,5212	0,7719
24. Ao tomar decisões, você teve tempo suficiente para que suas dúvidas fossem consideradas e suas perguntas respondidas?	0,3712	0,7989
25. Qual das seguintes opções melhor descreve sua opinião:	0,3369	0,8048
26. Durante as últimas horas de vida do seu familiar, qual das seguintes opções melhor descreve sua opinião:	0,5815	0,7607
27. Nas últimas horas antes do falecimento do seu familiar, qual das seguintes opções melhor descreve sua opinião:	0,5830	0,7604

*r - correlação; α - alfa de Cronbach.*

Considerando-se os 29 itens do questionário, o coeficiente alfa de Cronbach retornou ao valor de 0,93. Ao realizar as correlações (r) entre itens e o alfa de Cronbach (caso o item fosse excluído), os itens 2a, 2b e 2c continuaram apresentando correlação abaixo de r ≤ 0,30, sendo 0,2509, 0,2501 e 0,2634, respectivamente.

## DISCUSSÃO

O FS-ICU 24R, reformulado em 2018, possibilita que os profissionais da UTI avaliem a prática assistencial a partir da avaliação da satisfação dos familiares^(6)^. Embora apresente discretas modificações quando comparado ao FS-ICU 24, os autores recomendam a utilização da nova versão. Todavia, após revisão de literatura, não se evidenciou a adaptação da nova versão para outras culturas, o que limita a comparação desta discussão.

A utilização do questionário no Brasil depende, inicialmente, do processo de adaptação transcultural, que atendeu ao objetivo de realizar adaptação transcultural e validar o conteúdo do FS-ICU 24R para uso no Brasil, o qual demonstrou equivalências satisfatórias e média de IVC adequada em todos os quesitos. Esta avaliação torna-se relevante para se obter as equivalências necessárias para a aplicabilidade e reprodutibilidade do questionário^(24)^ que, neste caso, destina-se ao idioma português do Brasil.

A adaptação de instrumentos exige uma metodologia rigorosa e consistente, de modo a garantir as qualidades linguísticas entre a versão original e a traduzida^(25,26)^. Na etapa da tradução, evidenciaram-se discrepâncias relacionadas ao estilo das duas tradutoras: uma apresentou uma versão literal e informal, e outra adequou à melhor compreensão no português. Logo, buscou-se a equivalência semântica em cada item em comparação com a versão original, em um minucioso processo de comparação e modificação, conforme recomendação da literatura clássica^(16,21,22)^.

Evidenciou-se que o processo de adaptação é extenso e trabalhoso. Contém etapas sistematizadas que dependem de uma dedicação coletiva e, não obstante, participação ativa dos autores. Sobretudo, destaca-se o processo de tradução, pois não se trata de uma tradução simples e, evidentemente, é a partir dela que se desenvolveram as etapas seguintes.

A maioria dos estudos que adaptaram o FS-ICU 24 abordou a importância da tradução e retrotradução^(4,7-9,12,13)^, no entanto, apenas no Brasil^(4)^, Alemanha^(7)^ e China^(9)^, os autores detalharam a avaliação das equivalências, sendo a semântica apenas mencionada no estudo de adaptação ao português do Brasil da versão FS-ICU 24^(4)^.

Apesar da disseminação de estudos de psicometria, o rigor metodológico continua sendo um dos pilares a ser reforçado para sua correta utilização^(27)^, todavia ainda não se percebe homogeneidade e clareza neste processo. No que tange à especificação da literatura escolhida para o método, entre os estudos de adaptação do FS-ICU 24, apenas no Brasil^(4)^ e na Tailândia^(12)^, mencionaram atender às exigências de autores da psicometria clássica^(16,21)^.

No que tange à ordem das etapas, literaturas consolidadas seguem a avaliação de especialistas após a retrotradução^(16,17)^, mas este estudo esteve em consonância com a proposta de publicações brasileiras prévias^(4,18,19)^, mantendo a etapa de retrotradução após o comitê de especialistas, o que possibilita identificar erros de significado antes da retrotradução.

Em relação à autorização do autor principal, realizou-se contato via correio eletrônico, tal qual ocorreu nas adaptações realizadas em outros países como Brasil^(4)^, Coréia do Sul^(8)^, China^(9)^, Noruega^(11)^, Tailândia^(12)^ e Turquia^(13)^, em consonância com a literatura^(22)^, que recomenda autorização do autor principal do questionário original para realizar a adaptação e validação.

Na validade de conteúdo, verificou-se equivalência entre o questionário original e a versão em português, com média geral de 0,86 nas equivalências semântica, idiomática, cultural e conceitual, o que equivale a 80% de aceitação conforme recomendado^(23)^.

Neste cenário, a validade de conteúdo se mostrou confiável por contar com uma diversidade de profissionais, com distintas percepções e atuações junto ao paciente crítico e seus familiares. Destaca-se que, para tomada de decisão assertiva, os especialistas convidados à etapa de avaliação devem ter formação, qualificação e experiência na área do questionário avaliado, já que serão corresponsáveis pela versão pré-final^(24)^.

Para identificar inconsistências ou fragilidades no processo de tradução, recomenda-se a avaliação semântica com a população para a qual o instrumento se destina^(19)^. Priorizou-se a avaliação semântica nos diferentes níveis de escolaridade, conforme recomendações^(27)^, para obter compreensão do item pela população-alvo com menor grau de escolaridade. Supõe-se que a ausência de dificuldades na compreensão dos termos esteja relacionada ao fato de a maioria dos respondentes possuir elevado grau de escolaridade (graduação/pós-graduação).

Os resultados obtidos no pré-teste asseguram a capacidade de adequada adaptabilidade do FS-ICU24R para o idioma português brasileiro tal qual ocorreu nas adaptações do FS-ICU 24 no Brasil^(4)^, Alemanha^(7)^, Coréia do Sul^(8)^, China^(9)^, Irã^(10)^, Noruega^(11)^, Tailândia^(12)^ e Turquia^(13)^. Destaca-se que, nos estudos do Reino Unido, não foram realizadas as adaptações transculturais, visto que a língua nativa do questionário é o inglês, sendo apenas revisado pela população-alvo e profissionais de saúde^(28,29)^.

O alfa de Cronbach do FS-ICU 24R nas subescalas do questionário foi acima de 0,70 (α=0,89, α=0,91 e α= 0,79), demonstrando já no pré-teste elevada confiabilidade. Porém, a correlação dos itens 2a, 2b e 2c foi menor que 0,3. Desta maneira, de acordo com os resultados do pré-teste, esses itens necessitariam ser removidos; contudo, decidiu-se pela manutenção dos mesmos até que o FS-ICU 24R Brasil seja aplicado em uma amostra maior, como no teste de validação.

Ao considerar os 29 itens do questionário, o coeficiente alfa de Cronbach apresentou o valor de 0,93, o que representa um valor elevado. O número de itens afeta a consistência do instrumento^(30)^. Assim, questionários com muitos itens aumentam o valor do alfa de Cronbach, e isto não necessariamente implica um aumento da consistência interna do questionário; ao contrário, um valor baixo de alfa pode apenas significar que há poucos itens.

Estratégias utilizadas neste estudo, como de organização, detalhamento do processo e envolvimentos dos pesquisadores com as distintas etapas, permitem identificar itens “problemas”, de difícil consenso na tradução, de menor avaliação das equivalências e, consequentemente, refletidos na análise estatística com menor correlações (r) entre itens, como foi o caso dos itens 2a, 2b e 2c. Deste modo, o método bem apresentado oferece subsídios para o pesquisador observar cada etapa, no ensejo de obter clareza e identificar as fragilidades dos itens.

Dos estudos que adaptaram o FS-ICU 24, prevaleceram aqueles em que não se evidenciou avaliação preliminar das propriedades psicométricas^(4,8,9,11-13)^, o que impossibilita comparações entre os resultados. Neste estudo, o pré-teste foi realizado para verificação da compreensão dos itens em uma amostra piloto. Desta maneira, há de se ter cautela antes de firmar conclusões precipitadas em dados preliminares.

No Brasil, ainda impera o modelo de visitação restritiva^(31)^, semelhante a estudo latino-americano, com o máximo de seis horas por dia de envolvimento do familiar nas visitas^(32)^. Essa restrição das instituições na coparticipação, a partir da não adesão à visitação estendida, contribui para a percepção de não pertencimento do familiar, como observado em itens sobre a participação no processo de tomada de decisão e tempo suficiente para tomar decisão, os quais não representaram situações vivenciadas por alguns familiares dessa adaptação.

Na cultura chinesa, por exemplo, a família desempenha um papel fundamental na tomada de decisão, em que se predomina o respeito pelo idoso, com valores consistentes na responsabilidade familiar e os relacionamentos humanos harmoniosos^(9)^. O processo de adaptação do FS-ICU 24R no Brasil e na sequência sua validação poderá ser um precursor de comparação e análise da variabilidade cultural, sobretudo sobre as expectativas do familiar sobre o processo de tomada de decisão e a comunicação com a equipe da UTI.

### Limitações do estudo

Como limitação do estudo, destaca-se o fato de o pré-teste ter sido realizado com uma amostra selecionada por conveniência e ter na amostra a maioria dos familiares com, no mínimo, o ensino médio completo, o que pode ser justificado pela coleta de dados *online*, o que poderia distanciar aqueles com menor grau de escolaridade. Pretende-se, portanto, minimizar esta limitação na avaliação de suas propriedades psicométricas, já que a amostra será maior. Vale considerar que as respostas foram autorreportadas, e isso pode implicar viés de resposta e de memória, já que a coleta foi realizada há mais de um ano da internação. Logo, é possível que, ao aplicar este questionário a outras populações de familiares, não se observam os mesmos resultados.

### Contribuições para a área da enfermagem

A pesquisa contribui com a possibilidade de avaliar a satisfação de familiares de pacientes de UTIs, mediante o uso de um questionário adaptado para o português do Brasil, já que alcançou fidedignidade adequada no processo de adaptação transcultural proposto. Todavia, ainda se faz necessário verificar as propriedades psicométricas do FS-ICU 24R/Brasil, a fim de garantir a validade e confiabilidade.

## CONCLUSÕES

O desenvolvimento da versão FS-ICU 24R adaptada para o português do Brasil se mostrou de fácil aplicação e compreensão. Atendeu aos critérios de equivalência entre o questionário original e o traduzido, e seu conteúdo foi validado por especialistas. O pré-teste mostrou que o questionário apresenta consistência interna dos itens e, portanto, capaz de avaliar a satisfação das famílias dos pacientes de UTI.

Desse modo, conclui-se que o FS-ICU 24R/Brasil se encontra apto para ser testado quanto às suas propriedades psicométricas, a fim de garantir a validade e confiabilidade, o que poderá contribuir para o fortalecimento de pesquisas em instituições engajadas em envolver a família no processo de internação hospitalar, sobretudo em elaborar estratégias que assegurem a manutenção da satisfação da família durante a internação do paciente crítico da UTI.

O seguimento deste estudo, com a validação do FS-ICU 24R/Brasil, permitirá a comparação de resultados com distintas realidades, visto que as versões anteriores deste instrumento já foram validadas em diversos países, como Chile, Espanha e China, e vem contribuindo para uma reflexão sobre a participação da família nos cuidados e na tomada de decisão nesses serviços, favorecendo a qualidade do cuidado. Dessa forma, com a disponibilidade do FS-ICU 24R, espera-se identificar aspectos que precisam ser melhorados e aqueles que despertaram a satisfação do familiar e merecem ser mantidos e fortalecidos, além de contribuir na promoção de estratégias que impulsionem pesquisas sobre o envolvimento da família na coparticipação do cuidado e decisões.

## Data Availability

Os dados da pesquisa estão disponíveis no corpo do artigo.
